# An update on health literacy dimensions: An umbrella review

**DOI:** 10.1371/journal.pone.0321227

**Published:** 2025-06-10

**Authors:** Craig Smith, Stephen Behan, Sarahjane Belton, Catherine Nicholl, Maeve Murray, Hannah Goss

**Affiliations:** 1 Department of Public Health and Epidemiology, School of Population Health, Royal College of Surgeons in Ireland, University of Medicine and Health Sciences, Dublin D02DH60, Ireland; 2 School of Health and Human Performance, Dublin City University, Dublin, Ireland; Swiss Paraplegic Research, SWITZERLAND

## Abstract

Awareness of the value of health literacy has grown significantly in recent years, with the concept increasingly integrated within public health policy, research and practice, and notably, more commonly being explored in a systems approach to health promotion. The concept does however appear to be constantly evolving, with various iterations of definitions being used. As such, there is a need to review and update understanding of health literacy’s core components. This umbrella review aims to provide a contemporary understanding of health literacy and its dimensions. Searches were conducted between July and August 2023 across three databases, and 784 studies were identified and screened, with nine systematic reviews identified for extraction. All included systematic review articles aimed to conceptualise or define health literacy. Following a quality assessment, using the AMSTAR-2, inductive analysis was carried out to map specific health literacy dimensions identified in each included study to previously identified health literacy clusters. The findings of this umbrella review highlight the ever-evolving nature of the concept of health literacy; the responsibility placed on the individual to develop and maintain adequate health literacy. Despite the relatively poor reporting quality of many of the review articles included, the findings from this umbrella review point to the difficulty in developing tools to comprehensively measure health literacy, and critically, how to effectively improve health literacy at both an individual and populational level.

## Introduction

The concept of health literacy originated roughly 50 years ago [1], and over the past two decades, has started to garner considerable traction in research, policy, and practise due to its potential benefits to both individual and public health [2,3].

Originally, health literacy was predominantly concerned with the ability to read and write medical information, focusing mostly on applying general literacy skills to a health context [1]. As the understanding and application of the concept evolved, health literacy was positioned as a range of competencies required to understand, communicate, act upon health situations throughout life [4]. More recently, health literacy at a systems level has been advocated for, whereby an appreciation of the mediating effects of organisational structures and available resources on an individual’s health literacy has been considered [5].

In understanding health literacy at an individual level, Nutbeam [3] proposed that there are three ‘types’ of health literacy: functional, interactive, and critical health literacy. *Functional health literacy* is concerned with the basic reading and writing skills required to find health information and apply health knowledge to a range of prescribed activities and everyday situations. *Interactive health literacy* skills are more advanced cognitive social and literacy skills which can be used to participate actively in everyday situations, extract health information and derive meaning from different forms of health communication and apply this to changing circumstances. *Critical health literacy* is often considered the most advanced and is concerned with the ability to critically question health-related information in personal situations and to use this information to exert control over health situations. Given this typology, health literacy has been considered a set of skills that can be developed to provide one with greater autonomy and empowerment over their health decision-making. More recent work by Nutbeam & Lloyd [5] has considered health literacy as a social determinant of health, as evidence by a social gradient in health literacy in a range of national surveys [6].

Given the ever-growing impact of an individual’s social and physical environment on their health, as well as the increasing complexities of health care systems, the need for one to develop health literacy is at an all-time high [7]. This is further evidenced by multi-national data that highlights low health literacy levels across many countries in Europe, with 47% of participants showed to have limited levels of health literacy [6]. A trend that is also seen globally [8]. Low levels of health literacy have been shown to have many negative consequences, including increased likelihood of adopting poor behaviours, a higher risk of disease, decreased self-care, and reduced capacity to manage with illness and over- and misuse of health care systems [2,9]. The promotion of health literacy is therefore an important task in public health, with some stating that addressing health literacy on a national and international level should be a priority to reducing the prevalence of chronic diseases, early mortality and increasing health costs [10]. The World Health Organisation (WHO) has recognised health literacy as a critical determinant of health and committed to develop, implement, and monitor national and local strategies for strengthening health literacy in all populations and all educational settings [11].

In order to understand how best to develop, and monitor, health literacy, it is crucial to transparently understand what health literacy actually is at its core. As such, there has been a proliferation of reviews in this area that have sought to define the concept. Research carried out by Sørensen et al., [12] systematically reviewed existing definitions and concepts of health literacy within the international literature in order to develop a comprehensive and integrated definition and conceptual model. In the process, the authors identified a list of central dimensions and overarching clusters for each dimension to sit within. This was a seminal piece of research, which was crucial in developing the conceptualisation and understanding of health literacy, however, research is needed to update this work and detail any further growth of the concept and its constituent dimension(s) over the past 12 years.

Thus, this umbrella review aims to build on the methods and findings of Sørensen et al., [12] to provide a comprehensive update on the key dimensions of health literacy according to the international literature. The findings of this review are intended to provide clarity and support in operationalising the complex concept of health literacy, as well as informing future health literacy research, evidence-based practise and policy.

## Methods

This umbrella review was registered a priori with PROSPERO (ID: CRD42024535025) and followed the Preferred Reporting Items for Systematic Reviews and Meta-Analyses (PRISMA) umbrella review methodological guidelines [14]. See PRISMA checklist in S1.

### Study design

Given the existence of multiple studies which have previously aimed to define, conceptualise, or review definitions and conceptualisations of health literacy, instead of reviewing primary papers, an umbrella review [14], also known as a systematic review of reviews [15], was conducted. Umbrella reviews, which integrate the findings of previously conducted reviews on the same or similar topic, are a relatively new form of evidence synthesis. The comparison and contrasting of findings of review studies allows for the evaluation of the consistency of research findings, the compilation of greater volumes of evidence and the discovery of insights adding value beyond restating previous findings [16]. This type of review is particularly useful when multiple reviews already exist on a topic. Implementing an umbrella review in this instance allows for the contrast and comparison of different health literacy definitions and conceptualisations in order to develop a greater understanding and clarity of the concept, and to perhaps identify the most common health literacy dimensions. Although the systematic review by Sørensen et al., [12] reviewed definitions and conceptual models of health literacy and identified specific dimensions of health literacy, it is 12 years since this research was published with numerous studies focussing on the definition of health literacy published since that time.

### Search strategy

Given the purpose of this umbrella review, the search strategy adopted, and the databases searched within this study were based on a previous systematic review (Sørensen et al., 2012). Three electronic databases were searched between July and August 2023 and updated ahead of submission —PubMed, Web of Science and Scopus—to identify relevant evidence (please see S2 Appendix 1 for specific search terms). ‘English’ and ‘peer reviewed’ filters were marked on all searches and were limited to publications post 2012. All records were exported to Covidence systematic review software (Veritas Health Innovation, Melbourne, Australia; www.covidence.org) for screening, and all duplicates were removed. Two reviewers (CN, CS) independently assessed the eligibility of the studies. Following title and abstract screening, full-text copies of potentially relevant studies were obtained and screened for full-text inclusion. In the case of disagreement, a third author (HG) was contacted for discussion until consensus was reached.

### Study selection criteria

**Fig 1:**
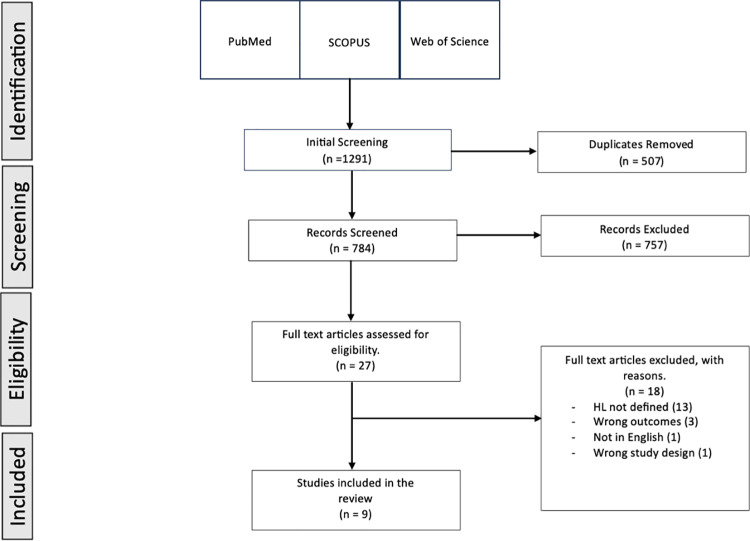
PRISMA flow chart.

The final review only included systematic review studies that (i) assigned primary focus to the definition of health literacy in the main body of the article, (ii) were published in an academic journal, (iii) were written in English language, and (vi) were published after 2012. We formulated no restrictions for the target population of the review. For more detail on the inclusion and exclusion criteria, see S3. Furthermore, see Fig 1 for PRISMA flow chart.

### Methodological quality

The quality of the included systematic reviews was assessed using Assessing the Methodological Quality of Systematic Review (AMSTAR) version 2 tool [17]. AMSTAR 2 is designed to evaluate systematic reviews that include randomised or non-randomised studies of healthcare interventions, or both and has been used in a number of similar umbrella reviews [17]. Given the nature of this umbrella review, some of the items within the AMSTAR-2 were not applicable (e.g., meta-analyses methods). Therefore, an adapted version of the tool was used within this study (see table 1 for all items assessed). The overall confidence was rated as high quality (if the review had no weakness or had only 1 noncritical weakness), moderate quality (if there were ≥2 noncritical weaknesses), low quality (if the review had only 1 critical weakness without considering noncritical weaknesses), or critically low quality (if there were ≥2 critical weaknesses with or without noncritical weaknesses). Further information about the critical and noncritical domains and the rating of the overall confidence is available elsewhere [17]. This process was completed by the lead author (CS), and the last author (HG).

**Table 1:** Qualitative assessment using the AMSTAR-2 tool.

Amstar-2 Item	Malloy-Weir et al., (2016)	Bröder et al., (2017)	Fleary et al., (2018)	Huhta et al., (2018)	Abedian et al., (2020)	Liu et al., (2020)	Truman et al., (2020)	Urstad et al., (2022)	Muscat et al., (2022)
Item 2: A priori protocol	N	N	N	N	N	N	N	Y	Y
Item 3: Study design	N	Y	Y	Y	Y	Y	Y	Y	Y
Item 4: Search strategy	N	PY	PY	PY	PY	PY	PY	PY	PY
Item 5: Study selection	N	Y	Y	Y	Y	Y	Y	Y	Y
Item 6: Data extraction	N	Y	Y	N	N	Y	Y	Y	Y
Item 7: Excluded studies	N	N	N	N	N	N	N	N	N
Item 9: RoB assessment	N	N	N	N	N	N	N	N	Y
Item 10: Reported funding	N	N	N	N	N	N	N	N	N
Item 13: Account for RoB in interpreting/discussing of results	N	N	N	N	N	N	N	N	N
Item 16: Conflict of interest	N	N	Y	Y	Y	Y	N	Y	Y
**Overall quality of the review**	CL	CL	CL	CL	CL	CL	CL	CL	CL

N = no, Y = yes, PY = partial yes, CL = critically low quality, RoB = risk of bias

### Analysis and synthesis of results

The methods adopted to analyse and synthesise the findings of this review were built on the work of Sørensen et al., [12]. Specifically, all studies included within the review were analysed and synthesised using a deductive content analysis (see S4 Fig 2). Firstly, eligible studies were screened for common definitions and conceptualisations of health literacy. Following this, a deductive analysis of the dimensions of each definition and/or conceptualisation of health literacy identified within this study was carried out using the six clusters of health literacy and the individual dimensions previously identified by Sørensen et al., [12]. These broader clusters of health literacy developed by Sørensen et al., [12] included [1] competence, skills, abilities; [2] actions; [3] information and resources; [4] objective; [5] context; and [6] time, with each individual cluster containing a range of individual dimensions relating to health literacy. Any dimensions of health literacy identified within the included studies were mapped to these clusters. Additional dimensions, which were not previously explicitly identified by Sørensen et al., [12] but were identified within the included studies, including synonyms where different wording is used to describe similar dimensions, were added to an appropriate wider cluster after consultation with the research team.

The aim of the deductive content analysis was to develop an updated comprehensive map of health literacy dimensions, each aligning to a specific cluster previously identified by Sørensen et al., [12].

## Results

An overview of the search process is provided in Fig 1. The literature search yielded 1291 publications; after removing 507 duplicates, 784 publications were subsequently screened. After screening of titles and abstracts, 27 studies were retrieved for full-text review. A total of 18 failed to meet the inclusion criteria, leaving nine remaining studies.

Table 1 details the quality assessment of the included review studies using the AMSTAR-2 tool. All studies included within the review were scored critically low quality as per Shea et al., [17]. Common quality lowering items were a lack of a priori protocol, lack of reporting funding in primary studies, lack of description of excluded studies, and the exclusion of a risk of bias tool.

## Health literacy dimensions

Please see Table 2 for a comprehensive table mapping the health literacy dimensions of each included study to those previously identified by Sørensen et al., [12]. Furthermore, the additional health literacy dimensions identified within this review are detailed. Each individual dimension has been mapped to the previously developed health literacy clusters (Sørensen et al., 2012). The number of times each dimension was identified within the review studies included within this review is also highlighted detailed within the table.

**Table 2:** Health literacy dimensions.

	Malloy-Weir et al., (2016)	Bröder et al., (2017)	Fleary et al., (2018)	Huhta et al., (2018)	Abedian et al., (2020)	Liu et al., (2020)	Truman et al., (2020)	Urstad et al., (2022)	Muscat et al., (2022)	Number of times dimensions identified
**Competence/ Skills/ Abilities**										
Skills		Y		Y	Y	Y			Y	5
Possession of requisite skills/Constellation of skills/Wide range of skills	Y			Y	Y		Y	Y		5
Personal skills				Y			Y			2
Cognitive skills	Y		Y	Y		Y	Y	Y		6
Social skills	Y		Y	Y		Y	Y	Y		6
The ability	Y		Y	Y	Y	Y	Y		Y	7
The capacity	Y	Y	Y	Y	Y		Y	Y		7
The competencies		Y			Y					2
The knowledge		Y		Y	Y	Y	Y	Y		6
Motivation	Y	Y	Y	Y	Y		Y	Y		7
Comprehension		Y				Y	Y			3
Communication	Y	Y			Y	Y	Y	Y		6
The strategies*		Y								1
Attitude*		Y		Y	Y			Y		4
Health practices*		Y			Y				Y	3
Confidence*				Y		Y				2
Self-efficacy*					Y	Y				2
Beliefs*				Y				Y		2
Self-directedness*					Y					1
Productivity*					Y					1
Interests*					Y					1
Self-awareness*					Y					1
Citizenship*					Y					1
(Environmental) Consciousness*					Y					1
Intention*					Y					1
Scientific knowledge*						Y				1
Education*							Y			1
Personal and economic abilities*							Y			1
**Action/ Agency**										
To understand	Y	Y	Y	Y	Y	Y	Y	Y		8
To comprehend	Y	Y			Y					3
To process	Y	Y		Y	Y	Y	Y	Y		7
To interpret					Y					1
To use	Y	Y	Y	Y	Y	Y	Y	Y		8
To act		Y		Y	Y					3
To perform basic reading and numerical tasks	Y		Y	Y	Y	Y	Y	Y		7
To perform arithmetic operations					Y					1
To read					Y	Y				2
To seek out	Y	Y		Y	Y	Y				5
To gain access	Y	Y	Y	Y	Y	Y	Y	Y		8
To find			Y	Y						2
To obtain	Y	Y		Y	Y	Y	Y	Y		7
To identify				Y						1
To filter				Y	Y					2
To appraise		Y	Y	Y	Y	Y	Y	Y		7
To evaluate	Y	Y	Y	Y	Y	Y				6
To communicate	Y			Y	Y	Y	Y			5
To embrace or disregard actions										0
To derive meaning						Y				1
To make sound decisions/to make health-related decisions				Y		Y				2
To take responsibility					Y	Y				2
To pertain interactions										0
To attain capacity, comprehension and communication							Y			1
To encompass*		Y			Y					2
To integrate*		Y								1
To construct*		Y			Y					2
To organise*		Y			Y					2
To create media messages*			Y							1
To navigate*			Y	Y	Y					3
To enable*			Y		Y					2
To function*				Y						1
To listen*				Y	Y					2
To recognise*				Y						1
To engage*				Y						1
To devise appropriate search strategies*				Y						1
To synthesise*				Y		Y				2
To manage*				Y	Y					2
To implement*					Y					1
To problem solve*					Y					1
To negotiate*					Y					1
To accept a condition/ learn to live with a disease*						Y				1
To cope*		Y			Y					2
To self-regulate*					Y	Y				2
To provide information*						Y				1
To set, initiate, manage and evaluate health goals*						Y				1
To apply*		Y	Y	Y	Y	Y	Y	Y		7
To employ*					Y					1
To acquire*			Y				Y			2
To think critically*					Y					1
To analyse*			Y	Y						2
To assess*				Y	Y					2
To discern the quality of information*				Y						1
To articulate*						Y				1
To decision make*				Y						1
To interact*		Y			Y	Y				3
**Information**										
Information	Y		Y	Y	Y	Y	Y	Y		7
Health information	Y	Y	Y	Y	Y	Y		Y		7
Information relating to health		Y		Y		Y	Y			4
Basic health information	Y	Y	Y	Y			Y	Y		6
Health-related print-material				Y		Y				2
Information presented in graphical form										
Health information in written, spoken or digital form						Y				1
Different forms of communication			Y			Y				2
Concepts	Y			Y		Y				3
Services	Y	Y	Y	Y	Y	Y	Y	Y		8
Health information from electronic sources*			Y	Y						2
Informational and communicative technology*			Y							1
Online information*				Y						1
Advertising and other media messages*			Y							1
Culturally relevant/ specific and age-appropriate information*					Y					1
Health data*					Y					1
Information relating to a healthy lifestyle*					Y					1
Health relevant terms, facts and principles*					Y					1
Medical and healthcare terminology, information, and instructions (medications, labels, appointments, forms, medical settings and systems)*				Y	Y					2
**Objective/ Purposes**										
Promote and maintain good health	Y	Y	Y	Y		Y	Y	Y		7
To promote health				Y	Y	Y		Y		4
To enhance health						Y				1
To improve health			Y	Y		Y				3
To function in the health care environment	Y				Y	Y				3
To make appropriate health decisions	Y	Y	Y	Y	Y	Y	Y	Y		7
To make informed choices	Y	Y		Y		Y	Y			5
To make appropriate health and care decisions				Y	Y	Y				3
To form sound judgments	Y	Y		Y	Y	Y	Y	Y		7
A critical empowerment strategy to increase people’s control over their health		Y				Y				2
Increase quality of life	Y	Y		Y	Y		Y	Y		6
To engage in demands of different health contexts										0
To make public health decisions that benefit the community										0
To accomplish health-related objectives										
Reduce health risks	Y	Y		Y						3
To influence individuals and society*		Y		Y			Y			3
To improve or achieve complete well-being*		Y								1
To achieve complete biopsychosocial health*					Y					1
To change their health behaviours or living conditions*		Y								1
To address or solve a health issue*			Y	Y						2
To build individual and community capacity to understand the components of health*		Y			Y					2
To understand that actions taken in youth affect health later in life*		Y								1
Understand health issues, such as drugs and alcohol*				Y						1
To be better able to understand others, themselves, and the world in a way that allows them to work on, and to make appropriate health decisions and change the condition that constitutes others’ health promotion chances and their own*					Y					1
To understand themselves, others and the world*		Y								1
To address or solve a health issue*			Y	Y						2
Disease prevention and treatment*				Y	Y		Y	Y		4
To modify their living conditions and health behaviour*					Y					1
To guide health actions*						Y				1
Staying healthy*				Y						1
Safety*				Y						1
First aid*				Y						1
Emergencies*				Y						1
Knowledge of appropriate treatment options*								Y		1
To facilitate recognition and treatment seeking*								Y		1
To better control health*					Y					1
To empower this group to be more engaged, more productive, and healthier*		Y			Y					2
To manage one’s health environment*		Y								1
**Context**										
The health care environment			Y	Y	Y	Y	Y	Y		6
Health care setting			Y			Y				2
Different health contexts										0
Health related contexts						Y				1
*The everyday life* at home, in the community, at the workplace, within the healthcare system, at the marketplace and within the political arena	Y			Y	Y	Y	Y	Y		6
Variety of settings	Y			Y		Y				3
HL always related to the context of the specific tasks needed to be accomplished										0
Physical and psycho-social activities/ biopsychosocial activities*		Y			Y					2
Online/ Internet*				Y						1
Health systems*						Y				1
Health environment*					Y					1
Health situations*				Y						1
**Time**										
Across the life course	Y	Y		Y			Y			4
Evolves over lifetime	Y						Y			1
Ongoing process*		Y								1
Starting at an early age*		Y								1

### Competences, skills and abilities

The wide range of skills associated with health literacy was highlighted within the review. Although many have stated vaguely that a health literate person requires personal skills or a constellation of skills [18–22], others have posited that one requires more specific cognitive and social skills. Examples of such, which are contained within the review, include the ability to comprehend [21,23,24] and communicate [18,20–24] appropriately, as well as someone’s level of knowledge [18,19,21–24]. Furthermore, the review underscored the psychological influence on health literacy. The importance of motivation was comprehensively reiterated across different reviews [18–23,25], as well as the specific influence of an individual’s self-efficacy [18,24], attitudes [18,19,22,23] and beliefs [19,22].

### Action and agency

This cluster included the widest variety of dimensions, with many new dimensions being added to the work of [13]. The ability to find, understand, critically appraise, and apply health information was clearly linked with the health literacy of a person, with each of these actions (or synonyms of these actions) occurring across all but one included review [26]. The significance of emotional regulation was also included as a key dimension within some reviews. For example, the ability to emotionally self-regulate to the demands of an experience and to cope with health situations or conditions [18,23,24]. Based on the findings of this review it is evident that basic literacy skills (such as reading, writing and communicating) are deemed critical to one’s health literacy [18–22,24,25].

### Information

Although the vast majority of included reviews identified information as being a key dimension to health literacy, how this was phrased varied across the studies. Some opted to just include ‘information’ broadly within their definition or conceptualisation [18–22,24,25], whereas many stated the information should be relating to health and health services [18–20,22–26] or health concepts [19,20,24]. Some studies were more specific, including the importance of being able to deal with medical and healthcare terminology, instructions, and information relating to health systems [18,19]. Additionally, the form of the information also varied, with some stating the importance of one not just being able to deal with health information in written and spoken form, but also emphasised the importance of dealing with digital, electronic, and online forms of information [19,24,25].

### Objectives and purposes

Similarly, this cluster was also wide-reaching, emphasising the important role of health literacy. The overarching purpose of health literacy, based on the included reviews, was to increase quality of life [18–23] and to promote or maintain health [18–25]. Other key objectives based on the findings of the review, included the ability to understand health issues and the components of health, and how to take action in order to address potential health complications and prevent disease [18,19,23]; the ability to make appropriate and informed healthcare choices, decisions and judgements [18–25]; to empower one to take more control over their health [18,23,24]; and to have the capacity to function within the healthcare environment [18,20,24].

### Context

Regarding the context, the healthcare environment was referred to most commonly within the review [18,19,21,24,25]. This includes health care systems [24], health settings [24,25], and health situations [19]. Many reviews described health literacy contexts as a ‘variety of settings’ and everyday life environments [19,20,24]. For example, in the community, workplace, marketplace, online, during physical and psychosocial activities and within the political arena [18–24].

### Time

The concept of health literacy, based on the review’s findings, applies across the life course, with some definitions and conceptualisations stating that it evolves over the lifetime.

## Discussion

This umbrella review has further highlighted the complex and multidimensional nature of the concept of health literacy, which is in line with previous literature in the area [13,19,23]. Building on the research of Sørensen et al., [12], this review has reinforced the wide-reaching nature of health literacy, with a considerable number of additional dimensions identified, specifically in relation to the competences, skills and abilities; action and agency; information; objectives and purposes; and context elements of the concept. To the best of our knowledge this is the first umbrella review carried out on this topic, providing a broad overview of the concept, and a contemporary update of the field of research to date.

This review presents evidence of the ever-developing understanding of the concept of health literacy. While the scientific debate and progression should be encouraged, a wide variety of understandings of what health literacy actually is will undoubtedly have a knock-on effect in the operationalisation of the concept. Within existing literature for example, organising components, consequences, antecedents of health literacy have been complex [9,27]. This is understandable considering the findings of this review, for example, many of the dimensions detailed within the various categories, particularly the ‘competences, skills and abilities’ and the ‘action/agency’ categories, have been deemed antecedents of health literacy in previous work [13,23]. Health literacy could be described as nebulous concept, whereby the perpetual growth of its dimensions and components can lead to difficulty in developing and delivering applicable health literacy interventions and educational material to support specific needs; in creating appropriate tools to measure health literacy; and in conducting health literacy related research to assess the impact of policy and practice. It must be noted, however, that this is a somewhat understandable development given the relatively recent emergence of health literacy as a concept. Health literacy has evolved from a basic definition to a more complex and diverse concept that encompasses an array of competences to meet the health needs of the modern world, as well as external factors that may play an influential role. It should also be considered that health literacy could, and arguably should, mean different things for different communities across the world [28,29]. It is vital that researchers, and others working in health literacy related areas, are clear and transparent in communicating how they define health literacy in the contexts they are working in. In addition, using the clusters to frame understanding could be a useful way of negotiating semantic differences across dimensions of health literacy (seen by the number of synonyms across the reviews). Notably however, this review only included English language studies, further research into how this approach may work across international definitions of health literacy would be useful.

This review not only highlights the complexity and potential importance of health literacy to empower individuals in modern society, but also the sizeable responsibility placed on an individual to develop and maintain adequate health literacy capacities. It is clear from the findings the immense number of competences, skills, and abilities, as well as the actions one may be required to acquire in order to navigate health-related situations across a range of contexts in everyday life. Such a skillset, coupled with the various and ever-growing number of antecedents to being health literate, such as the foundational competences (e.g., general literacy skills, individual characteristics) and demographic, psychosocial, and cultural factors [13], places a significant focus on the individual to develop their health literacy as an individual capability. In a recent publication within [30], the difficulty in making healthy choices, and the importance of moving away from the idea that health literacy is solely an individual’s responsibility was highlighted. The authors discussed how the health of an individual and wider communities is often impacted largely by the commercial determinants of health. This reiterates the findings of [9], who also highlighted that although socioeconomic and contextual factors were sometimes acknowledged in health literacy definitions and conceptualisations of young people, the majority of the emphasis is placed on the individual developing the health literacy capacities. Such an approach, which places the onus on the individual to develop the health literacy skills, fails to consider the range of factors that may be out of one’s control (e.g., social and commercial determinants of health) potentially acting as barriers to doing so.

Many more modern definitions of health literacy have adopted a multidimensional approach including critical health literacy dimensions from the work of [13] or adding their own dimension relating to health literacy domain. The importance of critical health literacy is echoed in wider research. A recent review study, however, stated that there remains a tendency for research to refer to early definitions of health literacy, which perhaps represent a more functional understanding of the concept, often devoid of the critical health literacy dimensions [19]. It has been suggested that this has had a knock-on effect on health literacy intervention design and delivery, whereby critical health literacy is often neglected [31]. Critical health literacy is often viewed as both one’s ability to critically appraise health-related information and as an individual’s capacity to act to improve health through the socio-political system. Therefore, critical health literacy is of great importance with regards to participating in critical dialogue and in decision making for health [31]. Thus, the importance of developing this domain of health literacy in order to improve health on an individual level and population level is evident. Despite this, continuing to use definitions which do not fully incorporate or emphasise the importance of critical health literacy has the potential to reduce the likelihood that this potentially vital component of health literacy will be incorporated into policy and practise. Future research work should be mindful of this if we want to advocate for a whole of society approach to health literacy.

It must be noted that there are a few limitations to this umbrella review. While studies did well in study design, selection, data extraction and declaring any conflicts of interest, the overall quality of the articles included scored as critically low as per the [17]. Future systematic reviews need to consider the reporting of all areas (i.e., AMSTAR-2), and this need should also encourage the transparency of reporting in primary published papers. Furthermore, the review only considered articles in the English language and did not screen grey literature. It is acknowledged that by excluding non-published articles, there is an increased risk of publication bias and the possibility of missing out on additional information that a grey literature search may have provided. This study, however, is the first to review the health literacy dimensions since the seminal work of Sørensen et al., [12], providing an update on the rapidly evolving concept of health literacy and its dimensions. Furthermore, a comprehensive search strategy was adopted, and two authors independently screened the articles for eligibility.

## Conclusion

This umbrella review aimed to develop a contemporary understanding of the concept of health literacy. Specifically, the study aimed to build on the work of Sørensen et al., [12] to provide an update on the key dimensions of health literacy according to the international literature. The findings from the review highlighted the ever evolving and multidimensional nature of the concept of health literacy, with many additional dimensions being added to the previously identified health literacy clusters. Furthermore, although the review highlights the importance of health literacy to empower individuals in modern society, it also emphasises sizeable responsibility placed on an individual to develop and maintain adequate health literacy levels. In existing definitions, there are an immense number of competences, skills, and abilities, as well as the actions, one may be required to acquire and apply in order to navigate health-related situations across a range of contexts in everyday life. It is, therefore, critical that there is an advocation for health literacy supports and education, but there should also be a focus on creating more health literate societies to facilitate better health choices. Finally, this review suggests some explanation for difficulties in operationalising health literacy in a meaningful and effective way. Nevertheless, despite these barriers, health literacy remains prevalent, and in order to continue this momentum future work should advocate for transparent, clear, and contextually relevant definitions. Ultimately, this should guide clear and effective action.

## Supporting information

S1 PRISMA Checklist. (DOCX)

S2 Search strategy. (DOCX)

S3 PICO Table (Inclusion/ Exclusion Criteria). (DOCX)

S4 Fig 2. The process of the deductive content analysis.
(DOCX)
