# Correction: An update on health literacy dimensions: An umbrella review

**DOI:** 10.1371/journal.pone.0332062

**Published:** 2025-09-10

**Authors:** Craig Smith, Stephen Behan, Sarahjane Belton, Catherine Nicholl, Maeve Murray, Hannah Goss

The first author, Craig Smith, is incorrectly noted as the corresponding author. The correct corresponding author is Hannah Goss. Hannah Goss’ email address is: hannah.goss@dcu.i.e.

An additional affiliation is missing for the first author. Craig Smith is also affiliated with the School of Health and Human Performance, Dublin City University, Dublin, Ireland.

## References

[1] Smith C, Behan S, Belton S, Nicholl C, Murray M, Goss H. An update on health literacy dimensions: An umbrella review. PLoS One. 2025;20(6):e0321227. doi: 10.1371/journal.pone.0321227 40493611 PMC12151441

