# Supplementary material for: An update on health literacy dimensions: An umbrella review
Source: PLoS One. 2025 Jun 10;20(6):e0321227. doi: 10.1371/journal.pone.0321227 (PMC12151441; doi:10.1371/journal.pone.0321227)
Supplement: S4 Fig — 2. The process of the deductive content analysis. (DOCX) [file pone.0321227.s004.docx]

**S4. Figure 2: The process of the deductive content analysis**

**
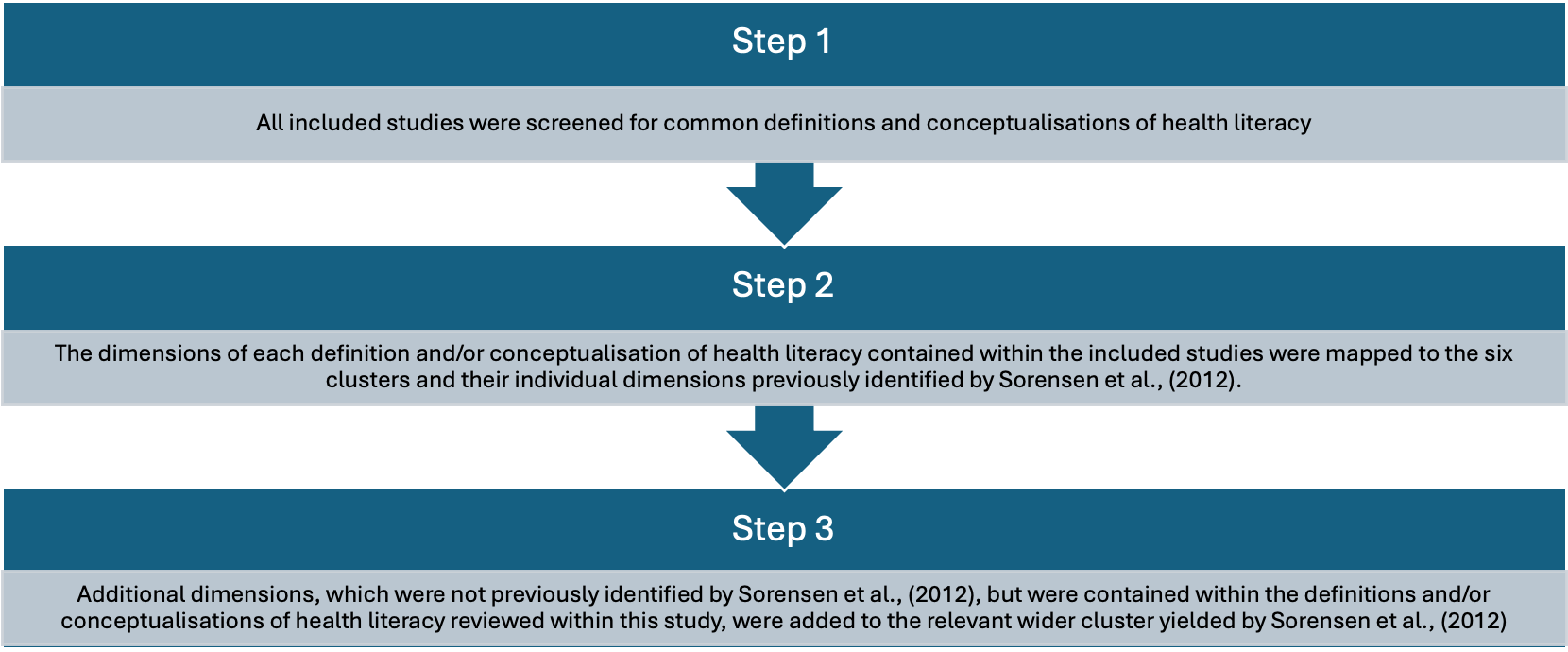
**

Figure 2. The process of the deductive content analysis
